# Emerging Plasma Technology That Alleviates Crop Stress During the Early Growth Stages of Plants: A Review

**DOI:** 10.3389/fpls.2020.00988

**Published:** 2020-07-15

**Authors:** Jong-Seok Song, Seong Bong Kim, Seungmin Ryu, Jaesung Oh, Do-Soon Kim

**Affiliations:** ^1^Plasma Technology Research Center, National Fusion Research Institute, Gunsan, South Korea; ^2^Department of Plant Science, Research Institute of Agriculture and Life Sciences, College of Agriculture and Life Sciences, Seoul National University, Seoul, South Korea

**Keywords:** atmospheric pressure plasma, seed germination, seedling growth, microorganism inactivation, antioxidant system, stress

## Abstract

Crops during their early growth stages are vulnerable to a wide range of environmental stressors; thus, earlier seed invigoration and seedling establishment are essential in crop production. As an alternative to synthetic chemical treatments, plasma technology could be one of the emerging technologies to enhance seed germination and seedling vigor by managing environmental stressors. Recent studies have shown its beneficial effects in various stress conditions, suggesting that plasma treatment can be used for early crop stress management. This paper reviewed the effects of different types of plasma treatments on plant responses in terms of the seed surface environment (seed scarification and pathogen inactivation) and physiological processes (an enhanced antioxidant system and activated defense response) during the early growth stages of plants. As a result, plasma treatment can enhance seed invigoration and seedling establishment by alleviating the adverse effects of environmental stressors such as drought, salinity, and pathogen infection. More information on plasma applications and their mechanisms against a broad range of stressors is required to establish a better plasma technology for early crop stress management.

## Introduction

**Figure 1:**
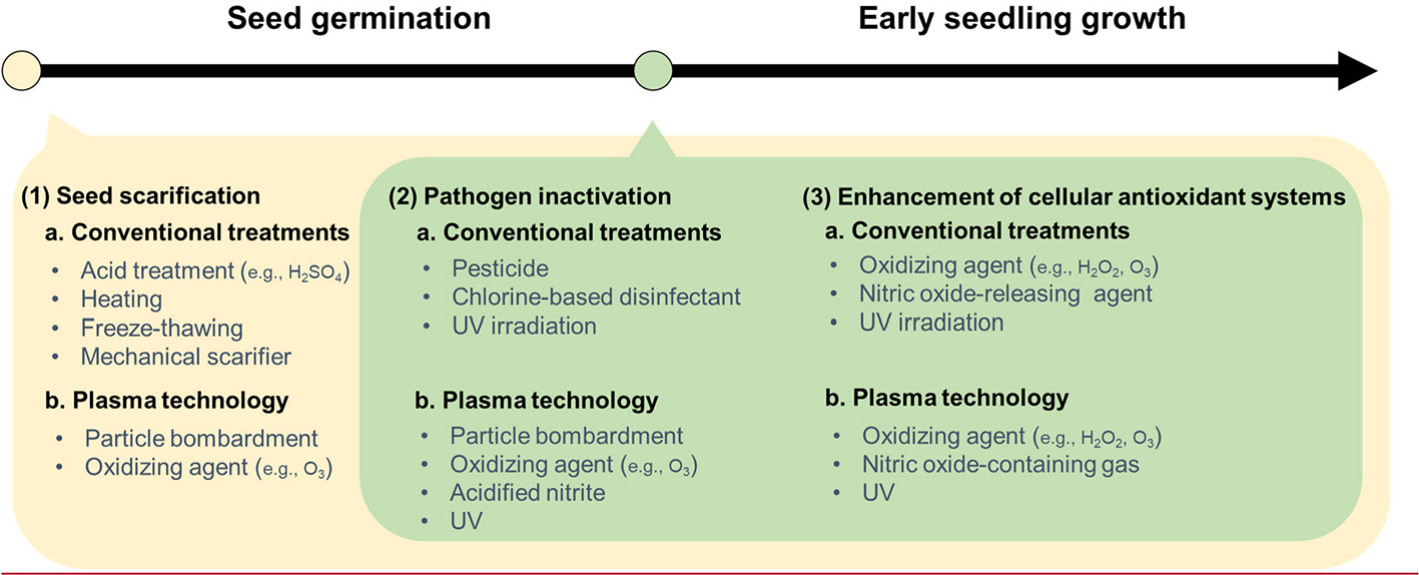
A comparison of plasma technology and conventional treatments with three factors affecting seed invigoration and seedling establishment: 1) seed scarification, 2) pathogen inactivation, and 3) enhanced cellular antioxidant systems. Seed scarification conventionally is done by acid treatment, heating, freeze-thawing, and mechanical scarifier. Plasma technology, as a particle bombardment or an oxidizing agent or both, can also be used for seed scarification at presowing. Additionally, plasma treatment, like other possible agents, can be applied at the early growth stage. Traditionally, synthetic chemicals have been commonly used for pathogen inactivation and antioxidant system activation.

Rapid seed invigoration and seedling establishment during the early growth stages of crops are necessary to prevent crop yield loss due to unfavorable environments. Seed germination and early seedling growth are the most sensitive growth stages for a crop to a wide range of environmental stressors (Ashraf and Foolad, 2005; Jisha et al., 2013; Sharma et al., 2015). Once the stressors affect the seeds or plants during the early growth stages, they can delay the onset, reduce the rate, and decrease the uniformity of germination and the emergence of seedlings. As a result, plant growth and final crop yield are reduced. Thus, seed invigoration is used to improve germination and seedling vigor (seedling size, health, and growth rate). Many efforts have been made to enhance seed germination and seedling vigor under both adverse and non-adverse conditions by applying various methods, including chemical treatments such as sulfuric acid, pesticides, and chlorine-based disinfectants (Ashraf and Foolad, 2005; Kimura and Islam, 2012; Jisha et al., 2013; Sharma et al., 2015) (see **Figure 1**). The synthetic chemical treatments are based on soaking seeds or spraying young plants using chemical substance-containing solutions. However, the use of synthetic chemical treatments can increase chemical pollutants in seeds or young plants and consequently can cause adverse effects on human health and the environment.

Plasma technology has been widely designated as an advanced oxidation process (Misra et al., 2011; Ekezie et al., 2017; Fan and Song, 2020). It has advantages over conventional treatments based on synthetic chemical substances, although large-scale applications of plasma technology are still expensive. A significant benefit of plasma technology is associated with its synergistic effects on seed germination and seedling vigor without any synthetic chemical residues. In general, an apparatus for plasma treatment simply consists of electrodes for plasma generation, a treatment chamber that accommodates the electrodes, and electric power to supply current to the electrodes. When a high electric discharge is applied to air or an aqueous solution in a chamber, reactive oxygen species (ROS, e.g., superoxides, singlet oxygens, atomic oxygen, ozone, hydrogen peroxide, and hydroxyl radicals), reactive nitrogen species (RNS, e.g., nitric oxide, nitrogen dioxide, nitrate, nitrite, and peroxynitrite), and ultraviolet (UV) photons are mainly generated from the plasma discharge (Selwyn et al., 2001; Laroussi and Leipold, 2004; Song et al., 2020). ROS, RNS, and UV have been independently used to scarify seeds (a technique to soften the seed coat while keeping the seed viable), inactivate seed-borne pathogens, and enhance antioxidant defense systems in crop plants (Jisha et al., 2013; Araújo et al., 2016; Antoniou et al., 2016; Thomas and Puthur, 2017). The synergistic effects of plasma treatment have recently been reported for a wide range of crops and reviewed by Randeniya and de Groot (2015), Ito et al. (2018), and Adhikari et al. (2020).

However, no review has yet summarized plasma applications and their protective mechanisms against a broad range of stressors. The potential applications of plasma technology for early crop stress management are largely unknown. This review evaluated the effects of the different types of plasma treatments on plant responses in terms of the seed surface environment (seed scarification and pathogen inactivation) and physiological processes (an enhanced antioxidant system and activated defense response) during the early growth stages of plants. Recent information on plasma applications and their mechanisms against a broad range of stressors was reviewed from these two perspectives. Thus, this review proposes that plasma technology has potential uses for seed invigoration and seedling establishment under stressful conditions.

## The Three Factors Affecting Seed Invigoration and Seedling Establishment

### Seed Scarification

The seed coat regulates seed germination through its thickness and permeability. Therefore, seeds with thick coats are incapable of water uptake rapidly unless it is scarified (Noodén et al., 1985). In particular, legumes (e.g., wild soybeans) have highly thickened and impermeable seed coats. In soybeans, a seed coat/embryo weight ratio higher than 0.1 indicates an impermeable seed coat (Noodén et al., 1985; Yaklich et al., 1986; de Souza and Marcos-Filho, 2001; Zhou et al., 2010). Therefore, seed scarification may often be required for seed invigoration and seedling establishment. **Table 1** summarizes the scarification effects of plasma treatments on seeds depending on the treatment conditions of the plasma sources, including radiofrequency (RF) discharge, dielectric barrier discharge (DBD), and other types of plasma sources. Power (W) and exposure time (minutes) are vital operating parameters to describe the beneficial effects among the individual elements of the plasma treatment (see **Supplementary Table 1** for additional factors).

**Table 1:** The scarification effects of the plasma treatments on seeds from a wide range of crops, including cereals, legumes, and vegetables.

Plasma	Treatment condition					Optimal condition	ROS/RNS ^c^	Crops	Effects ^a^	Maximum invigoration (%) ^d^	Reference
	Target stage	Medium	Feed gas ^b^	Power (W)	Exposure time (min)						
Radio frequency discharge	dry seed	gas	air	20	0–2	–	–	wheat	(+) germination % (+) time to germination	–	Bormashenko et al. (2012)
				50, 100	0–20	100 (W) × 7 (min)	excited N_2_	wheat	(+/-) germination %	3	Filatova et al. (2013)
				77–147	0–10	maize: 79 (W) × 2.5 (min); wheat: 79 (W) × 5 (min)	excited N_2_, NO, ^•^OH	maize, wheat	(+) germination % (+) growth %	Maize: 5; wheat: 12	Filatova et al. (2014)
				40, 60	0–20	60 (W) × 20 (min)	–	mung bean	(+) germination % (+) time to germination (+) growth %	40	Sadhu et al. (2017)
				20	0, 2	20 (W) × 2 (min)	–	common bean	(=) germination % (+) time to germination	–	Bormashenko et al. (2015)
	dry seed	gas	He	60–120	0, 0.25	80 (W) × 0.25 (min)	–	soybean	(=/+) germination % (=/+) time to germination (=/+) growth %	9	Li et al. (2014)
				100	0, 0.25	100 (W) × 0.25 (min)	–	oilseed rape	(+) germination % (+) time to germination (+) growth %	7	Li et al. (2015)
	dry seed	gas	C_6_H_5_NH_2_, C_6_H_12_	150	0–20	–	–	maize, soybean	(=) germination % (+) time to germination	–	Volin et al. (2000)
	dry seed	gas	N_2_H_4_	150	0–20	–	–	maize	(=) germination % (=) time to germination	–	Volin et al. (2000)
	dry seed	gas	CF_4_	150	0, 5	–	–	radish, pea	(=/-) germination % (-) time to germination	–	Volin et al. (2000)
	dry seed	gas	ODFD	150	0–20	–	–	maize, soybean, common bean	(-) germination % (-) time to germination	–	Volin et al. (2000)
Dielectric barrier discharge	dry seed	gas	air	2.7	0–30	2.7 (W) × 15 (min)	–	wheat	(=) germination % (+) time to germination (+) growth %	21	Dobrin et al. (2015)
				1.5	0–13	1.5 (W) × 7 (min)	O_3_	wheat	(+) germination % (+) time to germination (+) growth %	47	Li et al. (2017)
				370	0–10	370 (W) × 2 (min)	–	pea	(+/-) germination % (+/-) growth %	31	Stolárik et al. (2015)
	dry seed, wet seed	gas	N_2_ + air	400	0–1.3	400 (W) × 0.2 (min)	–	barley	(+) germination % (+) time to germination (+) growth %	51	Park et al. (2018)
	dry seed	gas	air, N_2_	–	0–5	5 (min)	excited N_2_	spinach	(+) germination % (+) time to germination (+) growth %	–	Ji et al. (2016)
Corona discharge	dry seed	gas	air	4.8	0, 1	4.8 (W) × 1 (min)	excited N_2_	rice	(+) germination % (+) time to germination (+) growth %	9	Khamsen et al. (2016)
Arc discharge	dry seed	gas	air	400	0, 0.0075	400 (W) × 0.0075 (min)	–	spinach	(+) germination % (+) time to germination	37.2	Shao et al. (2013)
Glow discharge	dry seed	gas	air	–	0, 10	10 (min)	–	rice	(+) germination % (+) time to germination (+) growth %	–	Chen et al. (2016)
			air, air + O_2_	60	0–15	60 (W) × 6 (min)	excited N_2_, ^•^O	wheat	(+) germination % (+) time to germination (+) growth %	25	Roy et al. (2018)
Microwave discharge	dry seed	gas	air	500	0–40	500 (W) × 5 (min)	–	wheat	(=) germination % (+) time to germination (=/+) growth %	5	Šerá et al. (2010)
			He	60–100	0, 0.25	80 (W) × 0.25 (min)	–	wheat	(=/+) germination % (+) time to germination (+) growth %	8	Jiang et al. (2014a)

a Plasma effects on the germination rate, time to germination, and growth rate of crops; the plus sign (+) indicates that the plasma treatment had a positive impact on the variable; the minus sign (-) represents that the negative effect of the plasma treatment increased, and the equal sign (=) indicates that a less-than-optimal plasma treatment did not generate a significant change on the tested variable.

b Feed gas, where He is helium; C_6_H_5_NH_2_ is aniline; C_6_H_12_ is cyclohexane; N_2_H_4_ is hydrazine; CF_4_ is carbon tetrafluoride; ODFD is octadecafluorodecalin; N_2_ is nitrogen; O_2_ is oxygen.

c ROS/RNS, where excited N_2_ is excited nitrogen species; NO is nitric oxide; ^•^OH is a hydroxyl radical; O_3_ is ozone, and ^•^O is an oxygen radical.

d Maximum invigoration (%) = (treatment-control)/control ×100.

RF discharge is extensively used to confirm plasma effects on seed invigoration of crops in a temperature-controlled chamber depending on the crop species, feed gas, power, and exposure time. Air and helium have been commonly used as feed gases for plasma generation driven by the RF discharge. RF discharge in air increased the seed invigoration of wheat, maize, and mung bean (Bormashenko et al., 2012; Filatova et al., 2013; Filatova et al., 2014; Sadhu et al., 2017). In the case of the common bean, a discharge in air reduced the time to achieve 50% germination, but it showed no significant change in the final percentage of germination (Bormashenko et al., 2015). Moreover, an excessive exposure of 20 min to an air-based discharge with a high power intensity of 100 W decreased the final seed germination of wheat (Filatova et al., 2013). RF discharge in helium improved seed invigoration with a 9% and 7% increase in the germination of soybeans and oilseed rapes compared with that of the untreated control, respectively, when generated with a power intensity of 80 to 100 W for an exposure time of 0.25 min (Li et al., 2014; Li et al., 2015). However, Li et al. (2014) reported that the exposure of a helium-based RF discharge with a power intensity lower than 60 W had no significant effect on the seed germination of soybeans. This result might be associated with the thick and impermeable testa (outer layer) of the soybean seed coat (Ma et al., 2004; Moïse et al., 2005; Shao et al., 2007). RF discharge has shown contrasting effects depending on the feed gases used. Volin et al. (2000) observed that RF discharge reduced the germination time of maize and soybean when generated from aniline (C_6_H_5_NH_2_) or cyclohexane (C_6_H_12_). However, RF discharge delayed the germination time of maize, radish, and some legumes such as soybeans, peas, and common beans when generated from carbon tetrafluoride (CF_4_) or octadecafluorodecalin (ODFD) otherwise known as perfluorodecalin (Volin et al., 2000). Additionally, RF discharge sometimes showed no significant effect on the germination time of maize when generated from hydrazine (N_2_H_4_) (Volin et al., 2000).

Dielectric barrier discharge (DBD) has been proven to be effective for seed germination and seedling vigor when generated under optimal conditions. DBD in air induced faster germination, leading to a better germination rate and early seedling growth of wheat, barley, and peas under laboratory conditions (Dobrin et al., 2015; Stolárik et al., 2015; Li et al., 2017; Park et al., 2018). A DBD with a power intensity of 1.5 to 2.7 W for an exposure of 7 to 15 min improved the seed invigoration of wheat by a maximum value of 47% compared with that of the untreated control (Dobrin et al., 2015; Li et al., 2017). In the case of a higher power intensity (370 to 400 W), DBD in air improved the seed invigoration by a 31% and 51% increase in peas and barley after an exposure of 2 and 0.2 min compared with that of the untreated control, respectively (Stolárik et al., 2015; Park et al., 2018). Additionally, DBD in nitrogen enhanced the seed germination and early seedling growth of barley and spinach (Ji et al., 2016; Park et al., 2018).

Other discharge plasmas, including corona, arc, glow, and microwave discharges, also have shown stimulating effects on seed invigoration of some crops although their maximum values differ depending on the treatment conditions of the plasma sources (Šerá et al., 2010; Shao et al., 2013; Jiang et al., 2014a; Chen et al., 2016; Khamsen et al., 2016; Roy et al., 2018).

**Figure 2:**
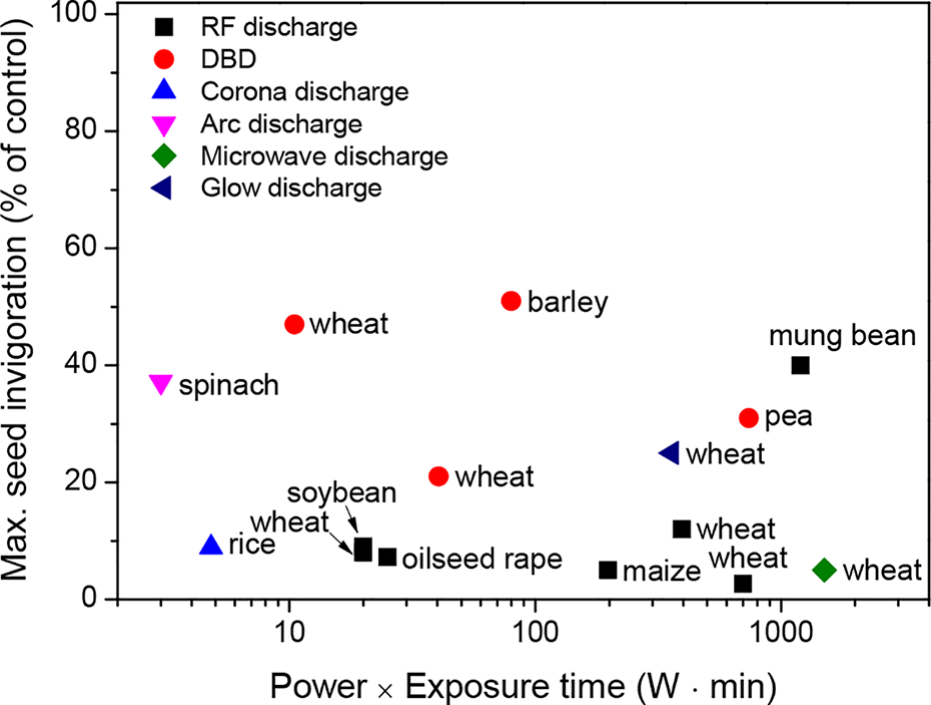
Maximum seed invigoration (% of control) as a function of the power (W) and exposure time (min) of the plasma treatments for a wide range of crops, which were described in the literature presented in **Table 1**.

These results suggest that plasma treatment can enhance the water absorption of seeds, thus leading to a better invigoration of crops. The beneficial effect of plasma treatment on seed invigoration is mainly associated with its power (W) and exposure time (min) (**Figure 2**). For example, the plasma treatment of seeds, when generated with a power of 100 W and an exposure time ≤ 10 min, can enhance the seed invigoration of crops without plasma-induced damage. These parameters for plasma treatment must be further optimized based on the crop species.

### Inactivation of Seed-Borne Pathogens

Pathogen inactivation by plasma treatment prevents plant diseases in crops (**Table 2**). In various studies, the power (W) and exposure time (min) are the most important determinants to inactivate seed-borne pathogens (see **Supplementary Table 2** for additional factors). In general, seed germination and early seedling growth are susceptible to plant diseases that are caused by seed-borne bacteria and fungi during crop growing seasons.

**Table 2:** The inactivation effects of the plasma treatments on seed-borne bacteria and fungi to prevent plant diseases in crops such as some cereals, legumes, and vegetables.

Plasma	Treatment condition					Optimal condition	ROS/RNS ^c^	Crops	Effects ^a^	Max. infection control (%) ^d^	Reference
	Target stage	Medium	Feed gas ^b^	Power (W)	Exposure time (min)						
Radio frequency discharge	dry seed	gas	air	77–147	0–10	77 (W) × 8 (min)	excited N_2_, NO, ^•^OH	maize, wheat	(+) fungal inactivation (*Alternaria* spp. and *Fusarium* spp.)	maize: 71; wheat: 99	Filatova et al. (2014)
			He	80	0, 0.25	80 (W) × 0.25 (min)	–	tomato	(+) bacterial inactivation (*Ralstonia solanacearum*)	25	Jiang et al. (2014b)
Dielectric barrier discharge	dry seed	gas	air	3	0–3	3 (W) × 10 (min)	–	rice	(+) fungal inactivation (*Gibberella fujikuroi*)	51	Jo et al. (2014)
				6.5	0–10	6.5 (W) × 5 (min)	O_3_	sweet basil	(+) fungal inactivation (*Alternaria* spp., *Aspergillus* spp., and *Penicillium* spp.)	44	Ambrico et al. (2017)
				400	0–10	400 (W) × 4 (min)	excited N_2_	wheat	(=/+) inactivation of filamentous fungi (*Fusarium nivale*, *F. culmorum*, *Aspergillus flavus*, and *Trichothecium roseum*)	100	Zahoranová et al. (2016)
	dry seed	gas	air, SF_6_	300	0–30	300 (W) × 20 (min)	–	wheat, chickpea, barley, oat, rye, lentil, maize	(+) fungal inactivation (*Aspergillus* spp. and *Penicillium* spp.)	–	Selcuk et al. (2008)
Arc discharge	wet seed	liquid	–	–	0–30	10–30 (min)	O_3_, ^•^OH, ONOO-	rice	(+) fungal inactivation (*Fusarium fujikuroi*)	–	Kang et al. (2015)

a Plasma effects on the inactivation of seed-borne pathogens; the plus sign (+) indicates that the plasma treatment had a positive impact on the variable, and the equal sign (=) indicates that a less-than-optimal plasma treatment did not generate a significant change on the tested variable.

b Feed gas, where He is helium, and SF_6_ is sulfurhexafluoride.

c ROS/RNS, where excited N_2_ is excited nitrogen species; NO is nitric oxide; ^•^OH is a hydroxyl radical; O_3_ is ozone, and ONOO- is peroxynitrite.

d Max. infection control (%) = (control-treatment)/control ×100.

Seed-borne pathogens cause plant diseases leading to reduced seed germination and seedling establishment of crops; however, RF discharge can inactivate seed-borne bacteria and fungi. The seed-borne fungi *Alternaria* and *Fusarium* species cause a range of economically significant diseases in a large variety of crops, including cereals, legumes, and vegetables (Thomma, 2003; Khan et al., 2006). In a vacuum chamber, an RF plasma treatment for 8 min with a power intensity of 77 W reduced fungal infection by the *Alternaria* and *Fusarium* species by a maximum value of 71% and 99% in maize and wheat, respectively (Filatova et al., 2014). With a similar power intensity of 80 W in a vacuum, an RF discharge inactivated the bacterial pathogen *Ralstonia solanacearum* after an exposure time of 0.25 min (Jiang et al., 2014b). *Ralstonia solanacearum* causes bacterial wilt symptoms on young tomato plants (Eljounaidi et al., 2016).

Other types of plasma sources such as DBD and arc discharge also have been shown to have inactivation effects on a wide range of filamentous fungi, thus resulting in a higher survival of crops. In the case of *Gibberella fujikuroi* (synonym, *Fusarium fujikuroi*), plasma treatments with DBD and arc discharge effectively inactivated its growth and thus reduced the fungal infection of rice by a maximum value of 51% under environmentally controlled conditions (Jo et al., 2014; Kang et al., 2015). Similarly, DBD in air effectively prevented the fungal infection of sweet basil in an *in vitro* seed culture from naturally established fungi including the *Alternaria*, *Aspergillus*, and *Penicillium* species by approximately 44% when generated with a power intensity of 6.5 W for an exposure time of 5 min (Ambrico et al., 2017). With a high power intensity of 400 W, DBD in air completely inhibited the growth of fungal pathogens, including *Fusarium nivale*, *F. culmorum*, *Aspergillus flavus*, and *Trichothecium roseum*, after an exposure of 4 min to wheat seeds *in vitro* (Zahoranová et al., 2016). Even in the case of a seed mixture, DBD treatment effectively reduced fungal contamination by the *Aspergillus* and *Penicillium* species (Selcuk et al., 2008). However, if no seed treatment is applied, fungal pathogens including the *Fusarium* and *Aspergillus* species and *Trichothecium roseum* are capable of inhibiting plant growth and the development of cereal and legume crops, thus resulting in severe crop loss (Tu, 1985; Parry et al., 1995; Goswami and Kistler, 2004; Khan et al., 2006; Klich, 2007; Palencia et al., 2010; Scherm et al., 2013). Additionally, the seed-borne *Fusarium*, *Aspergillus*, and *Penicillium* species produce plenty of mycotoxins that are potentially toxic to the health of both humans and animals (Kumar et al., 2008; Amaike and Keller, 2011).

**Figure 3:**
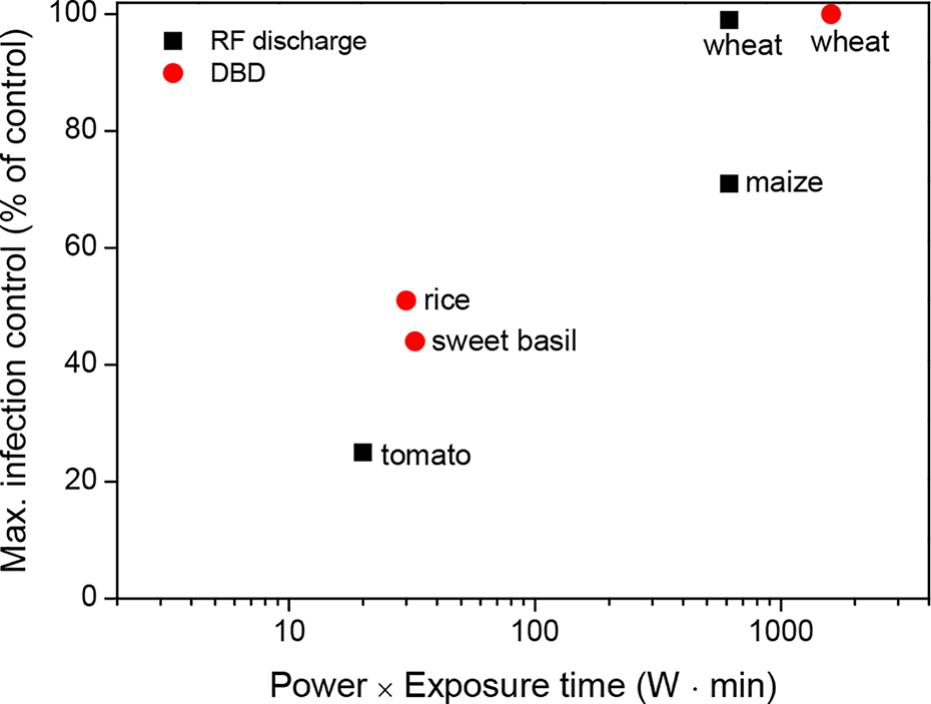
Maximum infection control (% of control) as a function of the power (W) and exposure time (min) of the plasma treatments for a few crops, which were described in the literature presented in **Table 2**.

Thus, plasma treatment of seeds can be used to prevent plant diseases in a wide range of crops, including cereals, legumes, and vegetables. According to **Figure 3**, plasma treatments might require a high power and long exposure to inactivate seed-borne pathogens effectively during seed germination. For example, a plasma exposure generated with a power of 100 W and an exposure time ≥ 10 min almost inactivates seed-borne pathogens in wheat. There are still other considerations when determining the standardized values for the power (W) and exposure time (min) of the plasma treatment because various parameters for pathogen inactivation can commonly occur depending on the environmental conditions.

### Enhancement of Antioxidant Defense Systems

Plants possess effective antioxidant systems to scavenge intracellular ROS and to protect cells against oxidative damage. Antioxidant defense systems consist of non-enzymatic components, such as quercetin and polyphenols, and enzymatic components, such as superoxide dismutase (SOD), catalase (CAT), and peroxidase (POD), in plant cells (Gill and Tuteja, 2010; Agati et al., 2012). The antioxidant enzymes SOD, CAT, and POD respond together when cells are exposed to excess ROS, although they work in different subcellular compartments (Mittler, 2002; Mittler et al., 2004; Sharma et al., 2012). SODs in almost all cellular compartments catalyze the dismutation (or partitioning) of the superoxide (O_2_^-^) radical into hydrogen peroxide (H_2_O_2_) and an ordinary molecular oxygen (O_2_). Finally, CAT in peroxisomes and PODs in the cytosol detoxify the hydrogen peroxide (H_2_O_2_) by catalyzing its reduction to water (H_2_O). Together with these enzymes, non-enzymatic antioxidants can provide cells with highly efficient machinery to detoxify the molecular species of active oxygen. After plasma treatment, the enhancement of these antioxidant systems has recently been reported in a few crops (**Table 3**).

**Table 3:** The enhancement effect of the plasma treatments on cellular antioxidant systems in crops, including cereals, legumes, and vegetables.

Plasma	Treatment condition					Optimal condition	ROS/RNS & UV ^c^	Crops	Effects ^a^	Enhanced enzyme activity (%) ^d^	Reference
	Target stage	Medium	Feed gas ^b^	Power (W)	Exposure time (min)						
Radio frequency discharge	dry seed	gas	He	80	0, 0.25	80 (W) × 0.25 (min)	–	tomato	(+) activity of antioxidant enzyme (POD)	100 (POD)	Jiang et al. (2014b)
				100	0, 0.25	100 (W) × 0.25 (min)	–	oilseed rape	(+) activity of antioxidant enzyme (CAT, SOD)	25 (SOD), 14 (CAT)	Li et al. (2015)
Dielectric barrier discharge	dry seed	gas	air	1.5	0–13	1.5 (W) × 4 (min)	O_3_	wheat	(+/=) activity of antioxidant enzyme (POD, SOD)	21 (POD), 22 (SOD)	Li et al. (2017)
				–	0, 4	1.5 (W) × 4 (min)	O_3_, NO_2_	wheat	(+) activity of antioxidant enzyme (CAT, SOD, POD)	92 (POD), 67 (CAT), 22 (SOD)	Guo et al. (2017)
				370	0–2	1 (min)	–	maize	(+) activity of antioxidant enzyme (SOD) (=/-) activity of antioxidant enzyme (CAT, POD)	–	Henselová et al. (2012)
	wet seed	gas	He, N_2_	100	0–2	100 (W) × 2 (min)	–	wheat	(+) activity of antioxidant enzyme (POD)	67 (POD)	Iranbakhsh et al. (2017)
	wet seed	gas	Ar	3.4–15.6	0, 0.2	15.6 (W) × 0.2 (min)	–	soybean	(+) concentrations of antioxidant enzyme (POD, CAT, SOD)	–	Zhang et al. (2017)
	dry seed	gas	air, N_2_	–	0–5	5 (min)	excited N_2_	spinach	(+/-) antioxidant content (total phenolic compounds)	–	Ji et al. (2016)
	dry seed	gas	air, Ar, N_2_	–	0–3	1 (min)	excited N_2_	coriander	(+/-) antioxidant content (total phenolic compounds)	–	Ji et al. (2015)
	seedling	gas	Ar	80	0–2	80 (W) × 1 (min)	^•^OH, UV	wheat	(+) activity of antioxidant enzyme (POD)	25 (POD)	Iranbakhsh et al. (2018)
Microwave discharge	dry seed	gas	air	500	0–40	500 (W) × 10 (min)	–	wheat	(+) antioxidant content (some phenolic compounds)	–	Šerá et al. (2010)
Glow discharge	dry seed	gas	air	–	0, 10	10 (min)	–	rice	(+) antioxidant content (total phenolic compounds)	–	Chen et al. (2016)
Arc discharge	dry seed	gas	air	400	0, 0.17	400 (W) × 0.17 (min)	–	tomato	(+) activity of antioxidant enzyme (POD)	100 (POD)	Yin et al. (2005)

a Plasma effects on cellular antioxidant systems of crops; the plus sign (+) indicates that the plasma treatment had a positive impact on the variable; the minus sign (-) represents that the negative effect of the plasma treatment increased, and the equal sign (=) indicates that a less-than-optimal or a more-than-optimal plasma treatment did not generate a significant change on the tested variable.

b Feed gas, where He is helium; N_2_ is nitrogen, and Ar is argon.

c ROS/RNS & UV, where O_3_ is ozone; NO_2_ is nitrogen dioxide; excited N_2_ is excited nitrogen species; ^•^OH is a hydroxyl radical, and UV is ultraviolet.

d Enhanced enzyme activity (%) = (treatment-control)/control ×100.

Cellular antioxidant systems are effectively enhanced by plasma treatments with a low power (W) and short exposure (min) (see **Supplementary Table 3** for additional factors). RF discharge, when generated with a power intensity of 80 to 100 W for a short exposure time of 0.25 min, rapidly increased the POD activity of tomato seedlings and the CAT and SOD activities of oil rapeseed seedlings when compared with the untreated control under environmentally controlled conditions (Jiang et al., 2014b; Li et al., 2015). Similarly, growing plants of wheat, maize, and soybeans had higher activities in the antioxidant enzymes POD, CAT, or SOD after DBD exposure to their seeds (Henselová et al., 2012; Guo et al., 2017; Iranbakhsh et al., 2017; Li et al., 2017; Zhang et al., 2017). In particular, DBD in air enhanced the POD and SOD activities of wheat seedlings after its exposure of up to 4 min with a power intensity of 1.5 W (Guo et al., 2017; Li et al., 2017). Wheat seedlings that were directly exposed to DBD for 1 min had a higher POD activity compared with the untreated control (Iranbakhsh et al., 2018). In the case of arc discharge, a short exposure enhanced the POD activity of tomato seedlings (Yin et al., 2005).

Dielectric barrier discharge has shown positive effects on the contents of the total phenolic compounds under laboratory conditions, although its effects depend on the types of feed gas. Ji et al. (2016) reported that an increase in the total phenolic compounds was observed in spinach seedlings after a nitrogen-based DBD treatment with an increasing duration of up to 5 min, while those treated with an air-based DBD and the same exposure time had a decrease in the total phenolic compounds. A similar study with micro DBD showed a higher content of total phenolic compounds in coriander seedlings after a nitrogen-based treatment up to 1 min (Ji et al., 2015). In contrast, a discharge in air increased the total phenolic compounds in the growing plants of wheat and rice when their seeds had a longer exposure time (Šerá et al., 2010; Chen et al., 2016).

**Figure 4:**
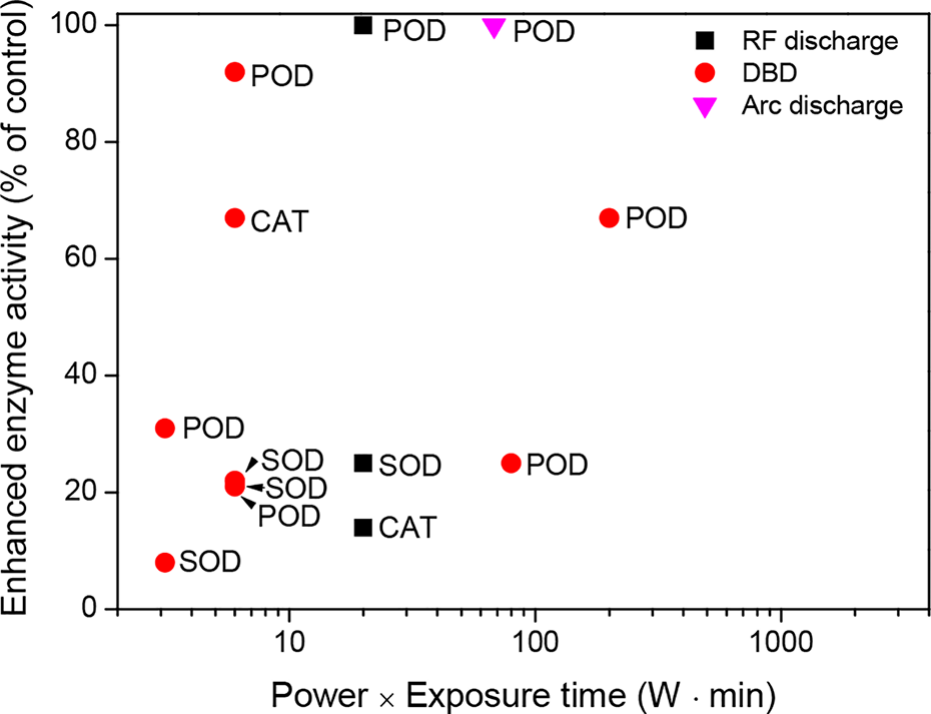
Enhanced enzyme activity (% of control) as a function of the power (W) and exposure time (min) of the plasma treatments in seeds (or seedlings), which were described in the literature presented in **Table 3**.

These results indicate that plasma treatments generated with a low power and short exposure time (for example, a power of 10 W and an exposure time ≤ 10 min) can enhance cellular antioxidant systems (**Figure 4**). In particular, antioxidant enzyme activities are increased by 8% to 100% compared to the untreated control. More experimental evidence is required to support the enhancement of the cellular antioxidant systems after plasma treatment.

## Combined Plasma Effects on Crop Stress Tolerance

### Enhanced Crop Tolerance to Environmental Stressors

Recent studies have suggested the potential beneficial role of plasma treatment on crop stress tolerance under environmentally controlled conditions (**Table 4** and **Supplementary Table 4**). The RF discharge treatment of seeds, generated with a power intensity of 40 to 100 W and an exposure time of 0.25 min, enhanced crop tolerance to drought stress in oilseed rape (Li et al., 2015) and alfalfa (Feng et al., 2018). Li et al. (2015) demonstrated that plasma treatment enhanced the water absorption of the seeds, increased the activities of antioxidant enzymes, and increased the accumulation of soluble sugars and proteins as osmolytes during stress. A similar study on RF discharge treatment reported plant disease resistance to *Ralstonia solanacearum*, which causes bacterial wilt in tomatoes. Jiang et al. (2014b) observed that plasma treatment induced a rapid increase in the H_2_O_2_ concentration, which sequentially increased the activities of antioxidant and defense-related enzymes in tomato leaves inoculated with *R. solanacearum*. In the case of DBD plasma, the optimal exposure also enhanced plant resistance to two seed-borne rice seedling diseases (bakanae disease and bacterial seedling blight) through H_2_O_2_-mediated signaling activating defense-related responses (Ochi et al., 2017). DBD treatment of wheat seeds increased the activities of antioxidant and defense-related enzymes, which suggests a potential role for plasma treatment in crop stress tolerance (Iranbakhsh et al., 2017). Guo et al. (2017) demonstrated that DBD treatment enhanced the water absorption of wheat seeds due to their surface modification. This modification alleviated drought-induced oxidative damage by regulating various biological processes (e.g., hormone-mediated signaling, drought-tolerant-related gene expression, antioxidant enzyme activation, and osmolyte accumulation). Interestingly, direct treatment of DBD on wheat seedlings also alleviated salinity-induced oxidative damage by inducing stress-related gene expression and activating antioxidant and defense-related enzymes (Iranbakhsh et al., 2018). Bußler et al. (2015) observed that repeated DBD treatment induced plant acclimation to other oxidative stresses (e.g., ROS, RNS, and UV) in peas in the early growth stage by increasing the concentration of flavonoid glycosides.

**Table 4:** The plasma effects on stress tolerance in crops.

Plasma	Treatment condition					Optimal condition	RONS & UV ^c^	Crops	Effects ^a^	Enhanced stress tolerance (%) ^d^	Reference
	Target stage	Medium	Feed gas ^b^	Power (W)	Exposure time (min)						
Radio frequency discharge	dry seed	gas	He	100	0, 0.25	100 (W) × 0.25 (min)	–	oilseed rape	(+) germination % (+) time to germination (+) growth % (+) water absorption (+) activity of antioxidant enzyme (CAT, SOD) (+) cell membrane stability (+) osmotic adjustment ability (+) tolerance to drought stress	6	Li et al. (2015)
				80	0, 0.25	80 (W) × 0.25 (min)	–	tomato	(+) germination % (+) time to germination (+) growth % (+) H_2_O_2_-mediated signaling (+) activity of antioxidant enzyme (POD) (+) activity of defense-related enzyme (PAL) (+) plant disease resistance to *Ralstonia solanacearum* (bacterial wilt)	25	Jiang et al. (2014b)
	dry seed	gas	air+ He	0–280	0.25	40 (W) × 0.25 (min)	–	alfalfa	(+/-) germination % (+/-) time to germination (+) growth % (+) tolerance to drought stress	40	Feng et al. (2018)
Dielectric barrier discharge	wet seed	gas+ liquid	air+H_2_O	–	0–10	10 (min)	–	rice	(+) growth % (+) H_2_O_2_-mediated signaling (+) plant disease resistance to *Fusarium fujikuroi* (bakanae disease) and *Burkholderia plantarii* (bacterial seedling blight)	–	Ochi et al. (2017)
	wet seed	gas	He, N_2_	100	0–2	100 (W) × 1 (min)	–	wheat	(+/-) growth % (+) activity of antioxidant enzyme (POD) (+) activity of defense-related enzyme (PAL)	–	Iranbakhsh et al. (2017)
	dry seed	gas	air	–	0, 4	4 (min)	O_3_, NO_2_	wheat	(+) germination % (+) time to germination (+) growth % (+) water absorption (+) hormone-mediated signaling (+) expression of drought-tolerant-related gene (*LEA1*, SnRK2, *P5CS*) (+) activity of antioxidant enzyme (CAT, SOD, POD) (+) cell membrane stability (+) osmotic adjustment ability (+) tolerance to drought stress	–	Guo et al. (2017)
	seedling	gas	Ar	80	0–2	80 (W) × 1 (min)	^•^OH, UV	wheat	(+/-) growth % (+) photosynthetic pigments (+) expression of plant HSF gene (+) activity of antioxidant enzyme (POD) (+) activity of defense-related enzyme (PAL) (+) tolerance to salt stress	20	Iranbakhsh et al. (2018)
	wet seed, seedling	gas	air	25	0–10	25 (W) × 5 (min)	O_3_, UV	pea	(+) germination % (+/-) growth % (+/-) photosynthetic efficiency (+) antioxidant content (total quercetins, total kaempferol) (+) acclimation to plasma-induced stress (ROS and UV radiation)	21	Bußler et al. (2015)

a Plasma effects on plant responses to stressors; the plus sign (+) indicates that the plasma treatment had a positive impact on the variable, and the minus sign (-) represents that the negative effect of the plasma treatment increased.

b Feed gas, where He is helium; N_2_ is nitrogen, and Ar is argon.

c RONS & UV, where O_3_ is ozone; NO_2_ is nitrogen dioxide; ^•^OH is a hydroxyl radical, and UV is ultraviolet.

d Enhanced enzyme activity (%) = (treatment-control)/control ×100.

**Figure 5:**
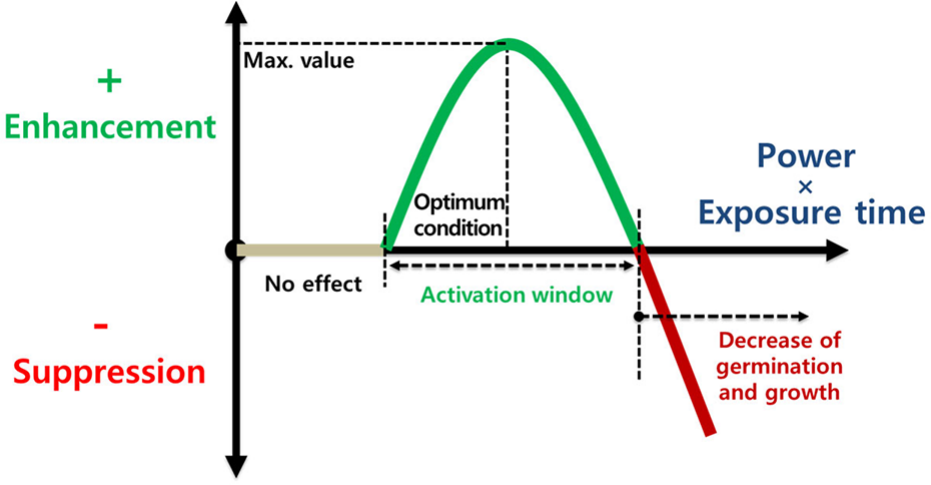
Crop response as a function of power (W) and exposure time (min) of the plasma treatment. The optimal plasma exposure can enhance seed germination and seedling growth before stress events.

**Figure 6:**
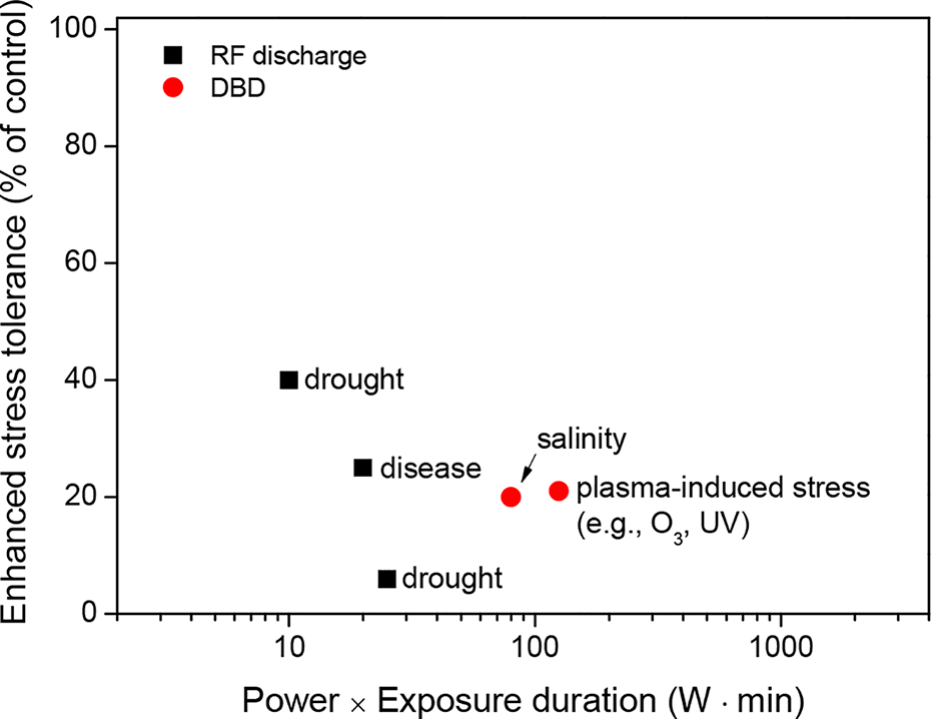
Enhanced stress tolerance (% of control) as a function of the power (W) and exposure time (min) of the plasma treatments on seeds (or seedlings), which were described in the literature presented in **Table 4**.

Thus, plasma treatments can enhance crop tolerance before stress events. The optimal plasma exposure can improve seed germination and seedling growth by modulating the seed surface environment (e.g., seed scarification and pathogen inactivation) and physiological processes (e.g., enhanced antioxidant systems and activated defense responses) in seeds under stressful conditions such as drought, salinity, and pathogen infection (**Figure 5**). In particular, only a plasma exposure with a low power and a short exposure time (for example, power of 10 W and an exposure time ≤ 10 min) can regulate plasma-induced crop tolerance (**Figure 6**).

### The Proposed Mechanisms for Stress Tolerance

**Figure 7:**
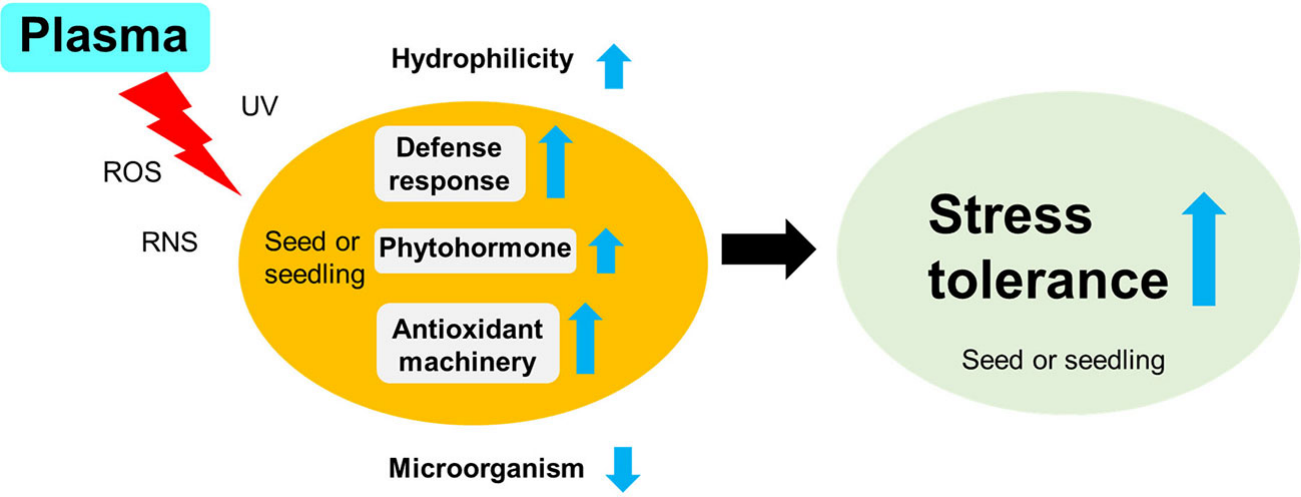
The proposed mechanisms of the enhanced stress tolerance to environmental stressors in plasma-treated seeds (or seedlings). The plasma treatment (e.g., ROS, RNS, and UV) can enhance crop tolerance before a stress event by modulating the seed surface environment (e.g., hydrophilicity and pathogen inactivation) and physiological processes (e.g., enhanced antioxidant system and activated defense response) in the seed (or seedling).

The plasma-induced stress tolerance might result from various interactions between the plasma (e.g., ROS, RNS, and UV) and the seed surface, seed-borne pathogens, and cellular homeostasis, respectively. Although the plasma-induced effects are still poorly understood, their potential mechanisms for crop stress tolerance can be suggested based on the review of the literature on plasma-induced plant responses (Section 2). The ROS, RNS, and UV of plasma can change the physical and chemical properties of the seed surface before exposure to environmental stressors, thus enabling seeds to be more hydrophilic and permeable to water. Scanning electron microscopy studies have shown partial degradation of cellulose and formation of cracks on the surface of plasma-treated seeds (Šerá et al., 2010; Mitra et al., 2014; Tong et al., 2014; Stolárik et al., 2015; Ji et al., 2016; Zhou et al., 2016; Li et al., 2017; Guo et al., 2018). Additionally, Fourier transform infrared spectroscopy and optical emission spectra studies have shown the formation of oxygen- and nitrogen-containing groups at the surface of treated samples (Filatova et al., 2013; Guo et al., 2017; Wang et al., 2017). Finally, plasma-treated seeds are hydrophilic and have cracks in the seed coat, which are beneficial for water uptake before exposure to drought stress (Li et al., 2015; Guo et al., 2017). Thus, the enhancement of water absorption can trigger faster germination and earlier seedling vigor by regulating endogenous hormones and hydrolytic enzymes during imbibition and sequentially supplying nutrients to an actively growing embryo (Li et al., 2014; Stolárik et al., 2015; Chen et al., 2016; Ji et al., 2016; Guo et al., 2017; Sadhu et al., 2017). Additionally, plasma-induced ROS, RNS, and UV can lead to better seed germination and seedling establishment under stressful conditions as follows: **1)** reduce infection with pathogenic microorganisms in germinating seeds or growing plants (Selcuk et al., 2008; Filatova et al., 2014; Jiang et al., 2014b; Zahoranová et al., 2016; Ono et al., 2017; Lee et al., 2019), **2)** regulate ROS homeostasis through the antioxidant machinery of plants (Yin et al., 2005; Šerá et al., 2010; Henselová et al., 2012; Bußler et al., 2015; Li et al., 2015; Chen et al., 2016; Ji et al., 2016; Guo et al., 2017; Li et al., 2017; Zhang et al., 2017), and **3)** activate other defense-related responses in plant cells (Iranbakhsh et al., 2017; Iranbakhsh et al., 2018). Many studies have shown that the application of exogenous ROS, RNS, or UV radiation has similar effects on seeds, thus resulting in an enhanced stress tolerance before stress events (Antoniou et al., 2016; Thomas and Puthur, 2017). Altogether, plasma-induced ROS, RNS, and UV could promote crop stress tolerance by regulating surface hydrophilicity, infection with pathogenic microorganisms, and diverse cellular mechanisms (**Figure 7**).

### Limitations and Future Considerations

Seed invigoration and seedling establishment under stressful conditions are highly variable depending on numerous factors, including biological (e.g., crop species, cultivar, and growth and developmental stages) and environmental factors (e.g., timing, duration, and intensity of the exposure to the stressor). There is little or no information for establishing crop stress management practices using plasma technology. To our knowledge, there are only a few studies under laboratory conditions on crop tolerance to disease (Jiang et al., 2014b; Ochi et al., 2017), drought (Li et al., 2015; Guo et al., 2017; Feng et al., 2018), salinity (Iranbakhsh et al., 2018), and oxidative stress (Bußler et al., 2015; Iranbakhsh et al., 2017) using plasma technology. Therefore, further studies are needed to optimize the effectiveness of plasma treatment on crop tolerance to a broad range of stressors. For example, flooding is highly detrimental to upland crops (Arduini et al., 2019; Arduini et al., 2020). Thus, plasma treatment could be useful to alleviate flooding stress, as demonstrated by a similar seed treatment using a magnetic field (Balakhnina et al., 2015). Future studies should extensively investigate plasma-induced stress tolerance under laboratory, greenhouse, and field conditions. Crop growth is greatly affected not only by biological and environmental factors but also by culture (e.g., irrigation, tillage, and fertilization) and plasma operating factors (e.g., plasma type, power, and exposure time). Further studies are necessary to determine the physiological, biochemical, and molecular mechanisms of stress tolerance in plasma-treated seeds or plants. More knowledge about the embryo (which is a young plant itself) within the seed is required to understand better the mechanisms of plant responses after plasma exposure. Specifically, a better understanding is necessary for the epigenetic changes and long-lasting plasma effects on the whole plant life cycle without any mutations.

## Conclusion

**Figure 8:**
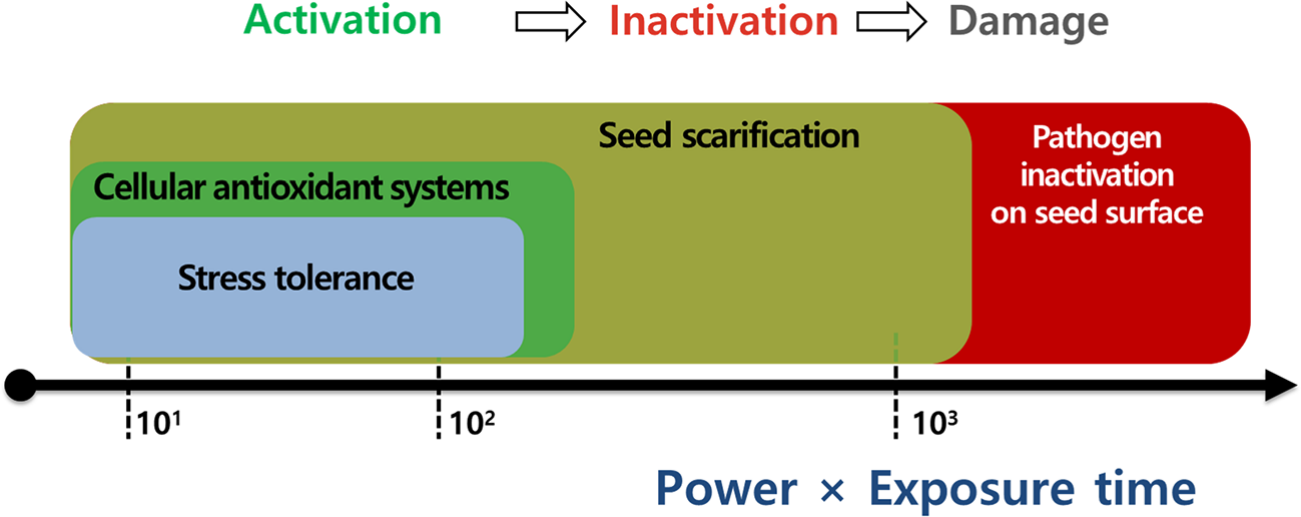
The most important parameters for plasma applications to enhance seed scarification, pathogen inactivation, cellular antioxidant systems, and stress tolerance in seeds or plants.

Plasma applications have been widely used for seed scarification, pathogen inactivation, and antioxidant system activation with proven beneficial effects on seed invigoration and seedling establishment under laboratory and greenhouse conditions. Moreover, the optimal plasma exposure can enhance crop tolerance before stress events (e.g., drought stress, plant disease, and oxidative stress) by modulating the seed surface environment (e.g., seed scarification and pathogen inactivation) and physiological processes (e.g., enhanced antioxidant system and activated defense response) in seeds. This promising technology is potentially valuable for alleviating the adverse effects of environmental stressors on seed germination and seedling growth in crop production. However, information on plasma effects under stressful conditions is limited to a few crops under laboratory conditions. Further studies are necessary to fully understand the effectiveness of plasma applications to a broad range of stressors under field and greenhouse conditions. This review suggests that the power (W) and exposure time (min) are vital operating parameters of the plasma treatment that affect the phenomenon of the plasma. More experimental evidence is needed to adopt the use of the power (W) and the exposure time (min) as standardized values for the plasma treatment of various crops (**Figure 8**).

## Author Contributions

J-SS conceived the idea for the topic, drafted and revised the manuscript, and initially provided all the Tables. SK helped draft the outline of the manuscript and provide most of the Figures. SR helped revise the Tables. JO provided **Figure 7**. D-SK helped draft the outline of the manuscript. All the authors critically reviewed the manuscript. All authors contributed to the article and approved the submitted version.

## Funding

This work was supported by the R&D Program of the “Plasma Advanced Technology for Agriculture and Food (Plasma Farming, Project No. EN2025)” through the National Fusion Research Institute of Korea (NFRI) funded by government funds.

## Conflict of Interest

The authors declare that the research was conducted in the absence of any commercial or financial relationships that could be construed as a potential conflict of interest.

## Abbreviations

RF, radiofrequency; DBD, dielectric barrier discharge; ROS, reactive oxygen species; RNS, reactive nitrogen species; SOD, superoxide dismutase; CAT, catalase; POD, peroxidase; PAL, phenylalanine ammonia lyase.
