# Supplementary material for: Emerging Plasma Technology That Alleviates Crop Stress During the Early Growth Stages of Plants: A Review
Source: Front Plant Sci. 2020 Jul 15;11:988. doi: 10.3389/fpls.2020.00988 (PMC7373780; doi:10.3389/fpls.2020.00988)
Supplement: Supplementary file 1 [file DataSheet_1.pdf]

Supplementary Table 1. The additional factors for the plasma treatments in the same literature presented in Table 1.

Plasma	Treatment condition			Reference
	Frequency (Hz) [a]	Pressure (Pa) [b]	Gas flow (L min^{-1})	
Radiofrequency discharge	10 M	6.7×10^{-4}	-	Bormashenko et al. (2012)
	13.56 M	66.65	-	Filatova et al. (2013)
	5.28 M	40–80	-	Filatova et al. (2014)
	13.56 M	20	-	Sadhu et al. (2017)
	10 M	6.7×10^{-4}	-	Bormashenko et al. (2015)
	13.56 M	150	-	Li et al. (2014)
	13.56 M	150	-	Li et al. (2015)
	13.56 M	9–40	-	Volin et al. (2000)
	13.56 M	9–40	-	Volin et al. (2000)
	13.56 M	9–40	-	Volin et al. (2000)
	13.56 M	9–40	-	Volin et al. (2000)
Dielectric barrier discharge	50	AP	1.0	Dobrin et al. (2015)
	50	AP	1.5	Li et al. (2017)
	14 k	AP	-	Stolárik et al. (2015)
	30	AP	3.0	Park et al. (2018)
	22 k	AP	1.5	Ji et al. (2016)
Corona discharge	700	AP	2.5	Khamsen et al. (2016)
Arc discharge	-	10–20	-	Shao et al. (2013)
Glow discharge	DC	800	-	Chen et al. (2016)
	3–5 k	1333	-	Roy et al. (2018)
Microwave discharge	2.45 G	140	2.5	Šerá et al. (2010)
	3 G	150	-	Jiang et al. (2014a)

[a] Frequency, where k is kilo; M is mega, and G is giga.
[b] Pressure, where AP is atmospheric pressure.

Supplementary Table 2. The additional factors for the plasma treatments in the same literature presented in Table 2.

Plasma	Treatment condition			Reference
	Frequency (Hz) [a]	Pressure (Pa) [b]	Gas flow (L min^{-1})	
Radiofrequency discharge	5.28 M	40–80	-	Filatova et al. (2014)
	13.56 M	150	-	Jiang et al. (2014b)
Dielectric barrier discharge	22 k	AP	-	Jo et al. (2014)
	500	AP	-	Ambrico et al. (2017)
	14 k	AP	-	Zahoranová et al. (2016)
	1 k	0.1–0.5	-	Selcuk et al. (2008)
Arc discharge	12	liquid	-	Kang et al. (2015)

[a] Frequency, where k is kilo, and M is mega.
[b] Pressure, where AP is atmospheric pressure.

Supplementary Table 3. The additional factors for the plasma treatments in the same literature presented in Table 3.

Plasma	Treatment condition			Reference
	Frequency (Hz) [a]	Pressure (Pa) [b]	Gas flow (L min^{-1})	
Radiofrequency discharge	13.56 M	150	-	Jiang et al. (2014b)
	13.56 M	150	-	Li et al. (2015)
Dielectric barrier discharge	50	AP	1.5	Li et al. (2017)
	50	AP	-	Guo et al. (2017)
	-	AP	-	Henselová et al. (2012)
	20	100	-	Iranbakhsh et al. (2017)
	60	AP	2	Zhang et al. (2017)
	22 k	AP	1.5	Ji et al. (2016)
	15 k	AP	1	Ji et al. (2015)
	20	100	-	Iranbakhsh et al. (2018)
Microwave discharge	2.45 G	140	0.2	Šerá et al. (2010)
Glow discharge	-	800	-	Chen et al. (2016)
Arc discharge	-	AP	-	Yin et al. (2005)

[a] Frequency, where k is kilo; M is mega, and G is giga.

[b] Pressure, where AP is atmospheric pressure.

Supplementary Table 4. The additional factors for the plasma treatments in the same literature presented in Table 4.

Plasma	Treatment condition			Reference
	Frequency (Hz) [a]	Pressure (Pa)	Gas flow (L min^{-1})	
Radiofrequency discharge	13.56 M	150	-	Li et al. (2015)
	13.56 M	150	-	Jiang et al. (2014b)
	-	-	-	Feng et al. (2018)
Dielectric barrier discharge	-	-	16	Ochi et al. (2017)
	20 k	100	-	Iranbakhsh et al. (2017)
	50	-	-	Guo et al. (2017)
	-	-	100	Iranbakhsh et al. (2018)
	3 k	-	-	Bußler et al. (2015)

[a] Frequency, where k is kilo, and M is mega.
